# Martial Arts as a Form of Undertaking Physical Activity in Leisure Time Analysis of Factors Determining Participation of Poles

**DOI:** 10.3390/ijerph15091989

**Published:** 2018-09-12

**Authors:** Elżbieta Biernat, Justyna Krzepota, Dorota Sadowska

**Affiliations:** 1 Department of Tourism, Collegium of World Economy, Warsaw School of Economics, 02-554 Warsaw, Poland; 2 Department of Physical Culture and Health Promotion, University of Szczecin, 71-065 Szczecin, Poland; justyna.krzepota@usz.edu.pl; 3 Department of Physiology, Institute of Sport—National Research Institute in Warsaw, 01-982 Warsaw, Poland; sadowska.dorota@hotmail.com

**Keywords:** martial arts, leisure time physical activity, factors, Poland

## Abstract

Background: The aim of this paper was to analyze selected sociodemographic and economic factors that determine practicing martial arts (MA) in Poland. Our hypothesis states that MA constitute a niche sport, which is a result of conditions shaping the decision to undertake them: perception through the lens of the media/entertainment business (rather than primary values), insufficient space for practicing, as well as high costs. Methods: The study was based on a survey conducted on a representative sample of Poles (n = 12,183). Results for 470 participants declaring some level of proficiency in MA were presented (including 124 declaring active participation). In order to verify statistically significant differences, a Chi-Square test, single-agent logistic regression analysis, and adds ratio were applied. Results: 3.0% of respondents declared basic skills in MA, while advanced skills were declared by 0.9% participants. Only 1% were active practitioners. The leading motivation was “pleasure” (62.1%), followed by “keeping fit and maintaining healthy body shape” (21.8%) and “health” (7.3%). The probability of participation in MA decreased with age (40 years of age being the turning point). As for young people, there is a 15 times lower chance of married individuals undertaking MA, while possessing advanced skills doubles the probability. School provides a place to practice MA during school years, but there is a problem with continuation at a later age (18.8% declaring classes organized at school vs. 5.4% organized at work). Conclusions: Reversing the trend of decreasing popularity of MA requires promotion on each level of education and creating opportunities to continue participation after graduation. There is a need to increase the availability of cheap sport facilities in the vicinity of the place of residence or work. In case of adults, it is important to build platforms facilitating contact with MA centers.

## 1. Introduction

The first decade of the 21th century saw a rapid development of mixed martial arts (MMA). According to Americans, MMA is the fastest developing sport [1] setting numerous attendances and viewing records [2,3], even more than boxing, which was previously regarded the most popular full contact sport. Considering the great number of fans of MMA or boxing in Poland and viewing figures for FEN 13 (*Fight Exclusive Night*), UFC 202 (*Ultimate Fighting Championship*) or KSW 36 (*Konfrontacja Sztuk Walki*) [4] night shows, it seems that martial arts (MA) in Poland are enjoying tremendous popularity [1]. This observation is supported by the results of the *ARC Rynek i Opinia* [5] survey that found boxing to be eighth among top the 10 sports in 2015 (with spectators reaching 3% of the Polish population and live transmissions watched by 48%). Although viewership of wrestling (2% of viewers), judo (1%), fencing (1%), taekwondo (1%), and karate (0.8%) [6] has declined in the last years, the world championships and the Olympic Games continue to attract combat sport enthusiasts.

MA are very spectacular for viewers, regarded as extreme, exotic, and brutal, which in the mass culture of consumerism represents a substantial attraction [7,8]. The MMA nights are made appealing by great live music and performances, highlighted by press conferences, staged meetings of fighting ring celebrities, and televised conferences. All of this usually goes to make blockbuster shows, which in the last years have risen in popularity [9]. These models, mass-produced by the media and film industry, tend to be highly influential [10]. However, there are also negative aspects of this phenomenon. Experts express concerns that the perception of MA only from the standpoint of media and entertainment may distort its real picture and ideas [11]. This popularization of the culture is likely to promote a culture of avoiding effort, banal entertainment that seeks brutality, strong excitement and spectacular shows, leading consequently to lose interpersonal contacts and passivity [7]. That is given a limited basis for comparison between the simulated fantasy and the real experience of MA training, and may lead members of the general public to form a number of preconceptions about the MA, some of which are accurate, many of which are not [12]. However, these collective assumptions do not only influence a person’s impressions of the MA, but may also motivate certain people to start practicing these sports [12]. Irrespective of the motivation, the pattern of warrior-hero is still popular [13].

Therefore, the question arises, how likely is this popularity of MA to translate into the active participation in the sports. Two Polish opinion polling institutes, *Centrum Badania Opinii Społecznej* [14] and *TNS Polska* [15], provided an unequivocal answer: boxing or other MA (e.g., karate, kung fu) are practiced by merely 2% of Poles. Similarly, the results of the survey conducted by *ARC Rynek i Opinia* [5] also showed a disproportion in viewing figures of boxing (56%) and participation in the sport (5%). It is worth examining why this happens if MA are so popular. Over 100 million people all over the world are involved in various forms of MA [16]. Researchers have demonstrated that they can be practiced at any age (since childhood until old age), regardless of gender or economic status [17]. The positive effect of MA on a person is multifaceted (they influence physical, mental, emotional, and spiritual development) [18,19]. MA give specific attention to many elements and differ from most other sports and physical activities, which usually focus on purely physical traits, in that they also emphasize exercises that harmonize body, spirit, and mind [20], teach relaxation, concentration, assertiveness, directiveness, and honesty in communication [21], and act as an agent that helps relax and reduce aggression [18,22]. Striving for a comprehensive individual development of a person, the skills learnt with MA training can turn out to be useful in cases of danger of a physical attack [23,24,25]. It is emphasized that they ensure health benefits connected with quality of life and preventing health problems [26,27] and perform a therapeutic function [28,29,30], for example in both “neurotic” and chronically mentally ill patients [21], children with intellectual disabilities [28], or individuals with multiple sclerosis (a kickboxing program) [30].

In light of the above facts, an analysis of participation of Poles in MA seems to be critical. Several questions arise, including: Who is practicing MA and where and how often are thy doing it? What is the sports skill level of people who participate in this form of physical activity? Who organizes training sessions and what are the costs of participation? To the best of our knowledge, there are few Polish scientific examinations in this field, although popularity of MMA has inspired researchers worldwide to identify the factors behind this phenomenon [31,32,33]. In two leading Polish scientific journals, *Archives of Budo* (archives since 2013) and *Journal of Combat Sports and Martial Arts* (archives since 2010), only one publication concerned the representatives of professional groups in Warsaw [19] and one of them presented a description of social, somatic, and psychological characteristics of wrestlers from the Polish National Cadet Team [34]. Therefore, it seems that enriching the insufficient information with quantitative examinations would enrich knowledge about the popularity of MA in Poland. Therefore, the aim of this study is to characterize selected sociodemographic and economic factors that determine practicing MA by Poles, with particular focus on the people who declare active participation.

## 2. Material and Methods

The study is based on the results of the survey ‘Participation of Poles in sport and physical recreation 2012’ (‘Uczestnictwo Polaków w sporcie i rekreacji ruchowej w 2012 roku’).

The survey was commissioned, designed, and carried out by the Central Statistical Office of Poland (CSO, *Główny Urząd Statystyczny*). Its ID number is DS-52. This paper-and-pen interview (PAPI) was originally designed to analyze the participation in sport and physical recreation as an indicator of the quality of life of Poles. The survey analyzed the aforementioned issues over the period of one year (October 2011 to September 2012) and is representative of the entire population of Poland.

Two questionnaires were used to capture both household and individual perspectives. The first questionnaire (DS-52 G) covered the characteristics of the household, sports equipment and expenditures, physical condition of its members, as well as barriers to sport and physical recreation (if an insufficient level was declared). Individual questionnaires (DS-52 I) were filled in by all household members, except for children aged 5–9, whose answers were provided by parents, and children below 5, for whom neither sport nor physical recreation was measured. The second questionnaire included individual skills, participation in training, description of the practice of selected disciplines, and motives for performing sport or physical recreation. Thirty-one groups of sports were distinguished, of which one was *martial arts* (*wrestling*, *judo*, *karate*, *boxing*, etc.). Moreover, a wide range of demographic and socio-economic characteristics was captured.

The sampling procedure in both surveys was based on a two-stage stratified sampling scheme with various probabilities of selection at stage one. At the first stage, the strata were field survey points representing six categories of place of residence, at the second-flats. The sampling frame was based on updated registers from the national census. The method of generalization included the probability of choosing the household based on the two-stage stratified scheme described above and census-derived data on the household structure by size (post-stratification by multiplication of two weights). The standard error estimation utilized the balanced half-samples (BRR) method.

The sample in the ‘*Participation …*’ survey was 12,405 individuals of which 12,183 answered the questions. In our paper, we decided to analyze all aspects related to *martial arts* (*wrestling*, *judo*, *karate*, *boxing*, etc.). Four hundred seventy individuals declared that they had skills related to the practicing of MA, with 360 people having basic level skills and 110 declaring advanced skills. Only 124 individuals who declared their skill level also reported practicing martial arts within the year analyzed. In this group, 99 individuals decided to include MA in the five chosen sports and elaborate more on their practice. In order to achieve the study aim, we measured martial arts participation and identified its determinants.

Despite the large sample size, the quantitative description of MA practice is challenging, as a small fraction imposes several restrictions on the potential methods. Due to this limitation, the paper is based mainly on descriptive statistics aimed at verification of relative and conditional frequency of MA practice as well as the distribution of practitioners’ characteristics. Descriptive analysis was completed with necessary statistical tests. The differences between particular categories were analyzed using the chi-squared test. Furthermore, a one-way analysis of logistic regression was performed and odd ratio (OR) was evaluated at 95% confidence intervals. The dependent variable was of Boolean dichotomous variable that adopted the value 0 in the case of the respondents who were non-practitioners of MA in the period analyzed and 1 if the respondent reported participation in MA over the period of the last year before the study. Statistical significance was set at *p* ≤ 0.05. Statistical analyses were performed using Statistica 12.0 software (StatSoft Inc., Tulsa, OK, USA).

Due to the topic of the survey and its anonymous nature, the research did not require an approval of the ethics committee.

## 3. Results

Of 12,405 randomly selected Poles, skills related to practicing MA were declared by 470 people (3.8%), including 360 respondents with basic skills and 110 with advanced MA skills. However, only 124 people (1% of all the respondents) declared that they had practiced the sports (either professionally or recreationally) in the period studied. The significant factors were age and gender of the respondents and the level of skills connected with the specific sports (Table 1). Having such skills at an advanced level nearly doubled the likelihood of taking up MA. Unlike single people, married Poles were almost 5 times less likely to practice such sports. Compared to children (5–14 years), this probability was 15 times smaller. Furthermore, the likelihood of participation in MA decreased with age. The analysis demonstrated that the 35% fraction of MA practitioners (people aged 10–19 years) falls rapidly to 21% at the age 20 to 29 years, to 18.5% at the age of 30 to 39 years, and to 5.6% at the age of ≥40 years. Therefore, it can be assumed that the age of 40 years represents the point of transition to inactivity in this respect.

Analysis of the group of Poles involved in MA (n = 124) revealed that most of them were people whose main motivation for participation in sports or physical recreation (in general) was pleasure and entertainment (62.1%) and keeping fit and having a healthy body shape (21.8%). The motivation of maintaining health or following the doctor’s recommendations concerned only 7.3% of the respondents from this group, whereas meeting friends, practicing sports or physical recreation, and other reasons concerned 4.0%, 2.4%, and 2.4%, respectively. No significant differences were found in these terms, neither with respect to sex nor place of residence of the respondents (cities/rural areas). A similar pattern was observed in the case of the subjective assessment of physical fitness. Among 124 people who practiced MA, 63.7% reported their fitness as very good, 29.0% as good, 6.5% as medium, and 0.8% declared bad or very bad fitness. Significant differences were found only in the level of education of practicing respondents (*p* = 0.016) depending on their sex (Table 2), and in income per one family member (*p* = 0.017) and character of work (*p* = 0.015) depending on the place of residence (Table 3). In the first case, compared to women, men who were involved in MA more often had secondary education (31.9% vs. 10.0%) and primary education (23.4% vs. 13.3%), whereas more women had tertiary education (26.7% vs. 17.0%) or were students (50.0% vs. 27.7%). Secondly, people involved in MA and those living in rural areas were more often from lower income groups (<600.00 PLN (PLN means new Polish zloty, the currency of Poland): 1.6% and 600.01–900.00 PLN: 24.3% vs. 5.8 and 12.6%, respectively) while their jobs more often required physical exercise (27.0% vs. 19.5%) compared to people living in cities. Furthermore, those who lived in cities had higher incomes more often (27.6% in the group of 1200.01–1700.00 PLN and 37.9% in those who earned >1700.00 PLN vs. 18.9% and 18.9%, respectively) and had sedentary jobs (20.7% vs. 2.7%).

Of 124 Poles who reported participation in MA, only 99 (79.8%) decided to characterize their participation in this form of activity in more detail. Table 4 presents detailed data. Unfortunately, due to a small size of this group, the analysis allowed only for the description of the frequency of choices made by respondents. The biggest percentage was found for the respondents who had practiced MA for 1 year (28.3%) and 2 years (28.3%). They had usually had training sessions 1 to 2 times a week on weekends (71.7%). The sessions were mostly organized by sports clubs (36.4%), schools (19.2%), and individual organizers (18.8%). Company programs offered such training sessions to 5.4% of the respondents. Although 12.8% of the respondents organized their MA sessions on their own, they were more often men (21.6%) than women (4.0%). For 49.5% of the respondents, average duration of the session was 1 to 2 h. The sessions occurred in sports centers (77.8%), with the commuting time of up to 59 minutes (78.8%). For 25.3% of the respondents, participation was free.

## 4. Discussion

Previous reports of the Central Statistical Office of Poland [35,36,37] pointed to a gradual increase in popularity of MA in Poland while documenting a gradually increasing number of sports clubs and people involved in such sports. Boxing was practiced by 2820 Poles in 2004, 3929 in 2010, with this number soaring to 5490 in 2014 (the number of boxing clubs also rose, with 71, 102, and 180, respectively). Furthermore, there were 7552 judo practitioners in 2004 and 7354 and 18,155 in the respective years (with the number of judo clubs at 82, 91, and 259, respectively). A similar pattern was observed for wrestling (number of athletes: 5297; 4577, and 7714, respectively, and the number of clubs: 96, 99, and 224, respectively), karate (number of athletes: 14,016; 18,314, and 22,125, respectively, and the number of clubs: 231, 244, and 345, respectively) and kick–boxing (number of athletes: 1384; 3218, and 5327, respectively, and the number of clubs: 28, 76, and 142, respectively). Similar trends were noted for such sports as taekwondo International Taekwon-do Federation (ITF) and World Taekwondo Federation (WTF), kendo, and ju–jitsu [35,36,37].

The present analysis indicates that nowadays, whatever the reasons, the popularity of MA is declining. MA are practiced by merely 3.8% of the Polish population. The survey by the Ministry of Sport and Tourism showed that this number was even smaller (3%) [38]. It should be noted that among 12,405 respondents, the involvement in MA was reported by 470 people (by making a choice out of 10 sports). However, as respondents went into more detail of the choices in the following questions, only a few (99 people) listed MA as one of five priority sports and described the character of making the decisions (in terms of time, frequency, place and the organizing entity, source of finance and commuting time). Although the questionnaire did not specify the criteria for selection of 5 of 10 sports, it can be presumed that respondents intuitively chose the sports that they practiced the most often. This leads to the conclusion that a specific role in popularity of MA among first 10 sports was played by incidental participation, caused by e.g. passive interest, but practice should involve concrete activity. This thesis can be supported by the respondents’ motivations for the involvement in physical activity: mainly ‘pleasure’ (62.1%), followed by ‘keeping fit and maintaining healthy body shape’ (21,8%), ‘health’ (7.3%), and meeting friends (4.0%). This is relatively different from previously described motivations (such as improved fitness, effective self-defense, and development of character [39] and values which MA were supposed to convey (psychophysical traits, self-improvement, integration, development of character traits, maintaining health, et al.) [16,17]. On the other hand, this is much closer to the media picture of MA, as emphasized by experts [7,40] and statistics that have shown the increasing popularity of spectacular consumer-focused forms of these sports [3,41]. Therefore, in order to avoid disappointment with such a dissonance, one should promote the primary image of MA [19,42]. The awareness of health benefits of MA should be also raised among Poles since of 124 people who practiced MA, 63.7% reported their fitness as very good, 29.0% as good, and only 0.8% reported bad or very bad fitness.

How can this decline in popularity of active participation in MA be reversed? It seems that the best action is to encourage children and young people to practice these sports at school and offer opportunities to continue them after finishing school. Data indicated that in the majority of countries MA are introduced during physical education classes in secondary schools (i.e., for pupils 10 to 14 years of age). Our study demonstrated a substantial percentage of young people (>10 years: 20.2%; 10–19 years: 34.7%) among practitioners, although this cannot be compared with China, where 13.7% of students are involved in regular MA training [43]. The inflow of adults to the group of MA practitioners is insignificant. Instead, gradual outflow is observed with age. Therefore, it seems justified to promote MA at all stages of education. Instilling the habit of regular training, development of a specific lifestyle (supported with knowledge about health benefits of such training and impact on intellectual and moral sphere) [44,45,46], and providing access to the sports infrastructure after finishing school may help change this situation. Many Polish professionals promote the implementation of elements of judo to the practice of sport and physical education [47,48,49]. Similar methodological approaches that present judo experts from Russia and Spain [50,51] are especially important since physical activity in childhood can determine healthy behaviors in adult life [52]. The correlations between physical education classes and the likelihood of the involvement in active forms of spending leisure time in the future are characterized by a feedback pattern [53].

It is essential that the space for exercising is ensured all year long. According to the Ministry of Sport and Tourism in Poland, the highest weekly intensity of playing soccer (3.7 h/wk) is linked to greater availability of soccer fields (especially those located in school facilities) or opportunities for practicing the sport in informal places such as lawns or meadows. Other sports, due to the lack of such opportunities, are practiced much less frequently [38]. Our results showed that school ensures the place for practicing MA over the period of education but the problem occurs with continuation of the activity into adulthood. This is not surprising as sports clubs and schools (with 36.4% and 19.2% of the respondents, respectively, training in such places) are natural forms of organization but designed only for young people. In adulthood, it is more difficult to take up sports in a sports club if one has never attended any, or to have contact with a school unless you have children at school. According to the most recent report by the Ministry of Sport and Tourism, although 40% of the respondents in Poland report the presence of a school gymnasium or a sports hall, they are convinced that access to such facilities can be difficult [38]. Adults do not feel welcome in such places. They considered school gymnasiums and sports halls as attractive and innovative places for younger people or those who are involved in team sports [38]. In the opinion of the survey participants, workplaces organize training programs only for an insignificant percentage of the respondents (5.4%). Therefore, it is important to provide access to sports facilities and to make using them and contact with sports clubs easier through creation of platforms for cooperation with workplaces and institutions that organize MA classes near the place of residence and workplaces. In our opinion, a 15-minute commute determines whether or not the person takes up MA. Actually, the group of Poles examined does not include those who practice MA and commute to training sessions to further places (the ministry reported the necessity of taking a half an hour walk) [38]. This is not surprising either in the case of children (problem for parents) nor in the case of adults (alternative cost of commuting). Longer commutes mean more time and higher expenses. It is very likely that this was also the cause of insufficient representation of the inhabitants or rural areas in the study. If availability has empirical importance, there should be more advertised places where people can practice. The costs are also critical. From our data, there are relatively few free classes. In a sense, this is obvious: it is necessary to rent a sports hall and pay for a coach (according to 18.8% of the respondents, the organizer is a private person). Furthermore, additional expenses have to be taken into consideration, such as buying sportswear or tickets. Therefore, MA are practiced more often by more wealthy adults (32.3% from the income group of >1700 PLN and 25.0% from the group of 1200–1700 PLN). Therefore, participation of children from relatively less wealthy families (not necessarily poor) is open to discussion. From the empirical point of view, this sport is not as exclusive as it may be in theory. Accordingly, the question remains how the relative costs of classes can be reduced, especially for children.

## 5. Conclusions

Increasing active participation in MA requires a systematic and programmed approach to promotion of these sports among children and young people. The approach should be not only quantitative but also qualitative. Instilling the values which can be conveyed by MA can lead to the establishment of healthy habits in adulthood. This means lifestyles where lifelong physical exercise is likely to represent an important component of developing character, improving health, quality of life, and prevention of the diseases of affluence [16,54,55,56,57]. These needs can be met by the increasing availability and access to inexpensive sports facilities near the place of residence or work. In the case of adults, it is important to build platforms that make it easy to integrate MA centers with various adult environments (workplaces, schools that children attend, sports clubs in community centers, etc.).

## Figures and Tables

**Table 1:** Factors determining practicing of martial arts in the Polish population (n = 470), odds ratios (OR) at 95% confidence interval (95% CI).

Factor	People Involved in Practicing Martial Arts		People not Involved in Practicing Martial Arts		β-Value	Odds Ratio (95% CI)	*p*–Value
	n	%	n	%			
Skill level							
basic	84	67.7	276	79.8	0.00	1.00	
advanced	40	32.3	70	20.2	0.63	1.88 (1.19; 2.97)	**0.007**
Place of residence							
rural areas	37	29.8	107	69.1	−0.05	0.95 (0.61; 1.49)	0.822
city	87	70.2	239	30.9	0.00	1.00	
Place of residence (according to the number of people)							
rural areas	37	29.8	107	30.9	0.00	1.00	
fewer than 20,000	9	7.3	25	7.2	0.04	1.04 (0.45; 2.43)	0.926
20 to 99 thousand	25	20.2	75	21.7	−0.04	0.96 (0.54; 1.73)	0.902
100 to 199 thousand	15	12.1	37	10.7	0.16	1.17 (0.58; 2.38)	0.660
200 to 499 thousand	14	11.3	40	11.6	0.01	1.01 (0.50; 2.07)	0.974
> 500,000	24	19.4	62	17.9	0.11	1.12 (0.61; 2.04)	0.713
Sex							
men	94	75.8	277	80.1	0.00	1.00	
women	30	24.2	69	19.9	0.25	1.28 (0.79; 2.09)	0.320
Age							
>10 years	25	20.2	9	2.6	2.31	10.02 (4.11; 24.43)	**0.000**
10–19 years	43	34.7	62	17.9	0.92	2.50 (1.37; 4.58)	**0.003**
20–29 years	26	21.0	71	20.5	0.28	1.32 (0.69; 2.52)	0.397
30–39 years	23	18.5	83	24.0	0.00	1.00	
≥40 years	7	5.6	121	35.0	−1.57	0.21 (0.09; 0.51)	**0.001**
Marital status							
unmarried	56	45.2	115	33.2	1.59	4.92 (2.76; 8.80)	**0.000**
married	18	14.5	182	52.6	0.00	1.0	
divorced, widow/widower	4	3.2	18	5.2	0.81	2.25 (0.69; 7.36)	0.181
students (5–14 years)	46	37.1	31	9.0	2.71	15.00 (7.72; 29.17)	**0.000**

The statistically significant results were presented in bold.

**Table 2:** Characteristics of Poles (n = 124) who reported practicing combat sports in the period studied (either professionally or recreationally) depending on sex and significance of differences based on the chi–squared test.

Characteristics of the Respondents		Total		Men		Women		*p*
		n	%	n	%	n	%	
Level of combat sports skills	basic	84	67.7	61	64.9	23	76.7	0.230
	advanced	40	32.3	33	35.1	7	23.3	
Place of residence (according to the number of people)	≥500,000	24	19.4	18	19.2	6	20.0	0.791
	200 to 499 thousand	14	11.3	11	11.7	3	10.0	
	100 to 199 thousand	15	12.1	13	13.8	2	6.7	
	20 to 99 thousand	25	20.2	17	18.1	8	26.7	
	<20,000	9	7.3	6	6.4	3	10.0	
	rural areas	37	29.8	29	30.9	8	26.7	
Place of residence	city	87	70.2	65	69.2	22	73.3	0.836
	rural areas	37	29.8	29	30.9	8	26.7	
Age	>10 years	25	20.2	16	17.2	9	30.0	0.061
	10–19 years	43	34.7	33	35.1	10	33.3	
	20–29 years	26	21.0	21	22.3	5	16.7	
	30–39 years	23	18.5	18	19.2	5	16.7	
	≥40 years	7	5.6	6	6.4	1	3.33	
Marital status	unmarried	56	45.2	46	48.9	10	33.3	0.186
	married	18	14.5	15	16.0	3	10.0	
	divorced, widow/widower	4	3.2	2	2.1	2	6.8	
	not applicable (5–14 years)	46	37.1	31	33.0	15	50.0	
Education	students	41	33.1	26	27.7	15	50.0	**0.016**
	primary	26	21.0	22	23.4	4	13.3	
	secondary	33	26.6	30	31.9	3	10.0	
	higher	24	19.4	16	17.0	8	26.7	
Character of work	sedentary	19	15.3	14	14.9	5	16.8	0.197
	requiring physical work	27	21.8	24	25.5	3	10.0	
	not applicable	78	62.9	56	59.6	22	73.3	
Money	we can afford to buy some luxury goods/money lasts us without saving	34	27.4	29	30.9	5	16.7	0.159
	money lasts us every day, but we have to save it to buy more expensive shopping	61	49.2	47	50.0	14	46.7	
	we have to spend money carefully/it lasts us for basic needs	29	23.4	18	19.2	11	36.7	
Income per family member	<600.00 PLN (<144.44 €)	13	10.5	12	12.8	1	3.3	0.297
	600.01–900.00 PLN (144.44–216.65 €)	20	16.1	13	13.8	7	23.3	
	900.01–1200.00 PLN (216.66–288.87 €)	20	16.1	17	18.1	3	10.0	
	1200.01–1700.00 PLN (288.87–409.24 €)	31	25.0	23	24.5	8	26.7	
	>1700.00 PLN (>409.24 €)	40	32.3	29	30.9	11	36.7	

The statistically significant results were presented in bold. PLN means new Polish zloty, the currency of Poland.

**Table 3:** Characteristics of Poles (n = 124) who reported practicing combat sports in the period studied (either professionally or recreationally) depending on their place of residence (cities/rural areas) and significance of differences based on the chi–squared test.

Characteristics of the Respondents		Total		City		Rural Areas		*p*
		n	%	n	%	n	%	
Skill level	basic	84	67.7	56	64.4	28	75.7	0.306
	advanced	40	32.3	31	35.6	9	24.3	
Sex	Men	94	75.8	65	74.7	29	78.4	0.836
	Women	30	24.2	22	25.3	8	21.6	
Age	<10 years	25	20.2	17	19.5	8	21.6	0.059
	10–19 years	43	34.7	27	31.0	16	43.2	
	20–29 years	26	21.0	16	18.4	10	27.0	
	30–39 years	23	18.5	21	24.1	2	5.4	
	≥40 years	4	3.2	6	6.9	1	2.7	
Marital status	unmarried	56	45.2	36	41.4	20	54.1	0.057
	married	18	14.5	16	18.4	2	5.4	
	divorced, widow/widower	4	3.2	4	4.6	0	0.0	
	not applicable (5–14 years)	46	37.1	31	35.6	15	40.5	
Education	students	41	33.1	27	31.0	14	37.8	**0.167**
	primary	26	21.0	16	18.4	10	27.0	
	secondary	33	26.6	23	26.4	10	27.0	
	higher	24	19.4	21	24.1	3	8.11	
Character of work	sedentary	19	15.3	18	20.7	1	2.7	**0.015**
	requiring physical work	−27	21.8	17	19.5	10	27.0	
	not applicable	78	62.9	52	59.8	26	70.3	
Managing money	we can afford to buy some luxury goods/money lasts us without saving	34	27.4	24	27.6	10	27.0	0.266
	money lasts us every day, but we have to save it to buy more expensive shopping	61	49.2	46	52.9	15	40.5	
	we have to spend money carefully/it lasts us for basic needs	29	23.4	17	19.5	12	32.4	
Income per family member	<600.00 PLN (<144.44 €)	13	10.5	5	5.8	8	21.6	**0.017**
	600.01–900.00 PLN (144.44–216.65 €)	20	16.1	11	12.6	9	24.3	
	900.01–1200.00 PLN (216.66–288.87 €)	20	16.1	14	16.1	6	16.2	
	1200.01–1700.00 PLN (288.87–409.24 €)	31	25.0	24	27.6	7	18.9	
	>1700.00 PLN (>409.24 €)	40	32.3	33	37.9	7	18.9	

The statistically significant results were presented in bold. PLN means new Polish zloty, the currency of Poland.

**Table 4:** Particular characteristics of participation in combat sports according to sex.

Characteristics of Participation in Combat Sports		Total		Men		Women	
		n	%	n	%	n	%
Frequency of participation in training sessions	occasionally (during holiday/leave)	7	7.1	6	8.1	1	4.0
	sporadically (once a month on average)	9	9.1	7	9.5	2	8.0
	once or twice a week/during leisure time at weekends	71	71.7	51	68.9	20	80.0
	3–5 times a week	11	11.1	9	12.2	2	8.0
	>5 times a week	1	1.0	1	1.4	-	-
Period of participation in training sessions	<1 year	9	9.1	6	8.1	3	12.0
	1 year	28	28.3	18	24.3	10	40.0
	2 years	28	28.3	23	31.1	5	20.0
	3–5 years	18	18.2	14	18.9	4	16.0
	6–9 years	8	8.1	6	8.1	2	8.0
	≥10 years	8	8.1	7	9.5	1	4.0
Mean duration of training sessions	<1 h	10	10.1	7	9.5	3	12.0
	1 h	33	33.3	25	33.8	8	32.0
	>1 h–2 h	49	49.5	38	51.4	11	44.0
	>2 h–3 h	6	6.1	4	5.4	2	8.0
	>3 h	1	1.0	-	-	1	4.0
Average commuting time	in the respondent’s place, without commuting	3	3.0	3	4.1	-	-
	≤59 min	78	78.8	57	77.0	21	84.0
	1 h–1 h 59 min	15	15.2	12	16.2	3	12.0
	>2 h	3	3.0	2	2.7	1	4.0
Monthly fee for participation in training sessions	no fees	25	25.3	21	28.4	4	16.0
	≤50 PLN (≤12.04 €)	33	33.3	23	31.1	10	40.0
	51–100 PLN (12.28–24.07 €)	32	32.3	23	31.1	9	36.0
	101–150 PLN (24.31–36.11 €)	4	4.0	3	4.1	1	4.0
	151–300 PLN (36.35–72.22 €)	4	4.0	3	4.1	1	4.0
	>300 PLN (>72.22 €)	1	1.0	1	1.4	-	-
Place of participation (facilities)	sports center	77	77.8	56	75.7	21	84.0
	fitness club/gym	16	16.2	13	17.6	3	12.0
	house/flat	3	3.0	3	4.1	-	-
	outdoors (park, forest, etc.)	3	3.0	2	2.7	1	4.0

PLN means new Polish zloty, the currency of Poland.
